# Motor cortex microcircuits

**DOI:** 10.3389/fncir.2013.00196

**Published:** 2013-12-12

**Authors:** Michael Brecht, Nicholas G. Hatsopoulos, Takeshi Kaneko, Gordon M. G. Shepherd

**Affiliations:** ^1^Bernstein Center for Computational Neuroscience, Humboldt University, and Cluster of Excellence NeuroCure, Charité-UniversitätsmedizinBerlin, Germany; ^2^Department of Organismal Biology and Anatomy, Committees on Computational Neuroscience and Neurobiology, University of ChicagoChicago, IL, USA; ^3^Department of Morphological Brain Science, Graduate School of Medicine, Kyoto UniversityKyoto, Japan; ^4^Department of Physiology, Feinberg School of Medicine, Northwestern UniversityChicago, IL, USA

**Keywords:** motor cortex, motor control, intracortical connectivity, corticospinal neurons, directional tuning

The goal of this Research Topic was to bring together articles representing the spectrum of current research aimed at understanding the functional organization motor cortex at the level of microcircuits.

The original research articles in this collection address a wide range of aspects of motor cortex microcircuits. The monkey's motor cortex is an especially important model system because of the similarities to the human brain, and the ability to train monkeys to perform complex movements. However, information about the cellular composition of different primates has been limited; Young et al. (2013) now describe the cell densities in motor cortex across multiple primate species. Studying reaching and grasping is a powerful approach to understanding complex movements in monkeys. Riehle et al. (2013) describe the spatio-temporal structure of motor cortical local field potentials and spiking activities during reach-to-grasp movements. Dickey et al. (2013) report on the heterogeneity of signals detected as monkeys make corrective movements while reaching. Motor cortical influences on lower limb function are also crucial for many types of motor behavior, and Hudson et al. (2013) report new findings of differences in the cortical output to fast and slow muscles of the ankle. The rodent motor cortex offers a complementary model system providing more immediate access to identified cells and circuits using optogenetic and related tools. In rats, Tanaka et al. (2011) dissect the local connectivity of corticospinal neurons with different classes of interneurons. Smith and Alloway (2013) show that the whisker motor cortex has distinct sensory-input and motor-output sub-regions. Applying optogenetic tools in mice, Hira et al. (2013) characterize the synaptic connectivity between rostral and caudal sub-regions encoding the forelimb representation. Studying genetically labeled pyramidal neurons in layer 5, Yu et al. (2008) demonstrate cell-type-specific local circuits and firing patterns. Also examining firing patterns, Hedrick and Waters (2012) report on their high sensitivity to temperature.

The review-type articles provide new syntheses of current knowledge about different aspects of motor cortex function and dysfunction. Kaneko (2013) focuses on microcircuits of excitatory neurons in the rodent motor cortex, and develops novel concepts about the organization of thalamic innervation to motor cortex microcircuits. Tsubo et al. (2013) assess current knowledge about *in vivo* dynamic activity across motor cortical layers in relation to movement. Harrison and Murphy (2012) emphasize the significance of particular classes of projection neurons and how these may be investigated with optogenetic strategies to determine their roles in motor function. Capaday et al. (2013) address the functional organization of the motor cortex from the perspective of intracortical connectivity. Castro-Alamancos (2013) discusses how motor cortex operates as a dynamic, frequency-tuned, oscillating network. Mahan and Georgopoulos (2013) review directional tuning from the perspective of resonance and the role of inhibitory mechanisms. Di Lazzaro and Ziemann (2013) review evidence, gathered from transcranial magnetic stimulation studies, for the roles of different types of microcircuits in the functions of human motor cortex. Diseases of the motor cortex have devastating consequences for motor control; Estrada-Sanchez and Rebec (2013) review the state of research on motor cortical involvement in Huntington's disease.

We are impressed not only with the diversity of contributions included here, but even more so we were delighted that researchers from all walks of motor cortex investigation enthusiastically steered their research toward the microcircuit theme pursued in this volume. More than ever it seems clear that we all are working toward a common goal, i.e., describing motor cortical function in terms of the transactions in identified cellular circuits. We thank the authors for their contributions, and are additionally grateful to the many reviewers who contributed their efforts.
